# Microstructural alterations of human medial temporal lobe in dementia: volumetric and DTI findings correlate with cell number and neuropathology

**DOI:** 10.1007/s00429-026-03223-4

**Published:** 2026-11-13

**Authors:** Emily Castro-Fonseca, Fernanda Meireles, Marina Monteiro, Daniel Menezes Guimarães, Viviane Morais, Jaqueline Freitas, Caroline V. Felix de Lima, Fernanda H. P. de Moraes, Ivanei Bramati, Rodrigo Vianna-Barbosa, Juliana Wollner, Camila Gomes da Silva, Arthur F. Mello-Neto, Luiz E. Oliveira, Carlos H. Andrade-Moraes, Ana V. Oliveira-Pinto, Felipe K. Sudo, Naima Assunção, Ana T. Alho, Renata E. L. Ferretti-Rebustini, Renata E.P. Leite, Claudia K. Suemoto, Roberta D. Rodriguez, Wilson Jacob-Filho, Ricardo Nitrini, Carlos A. Pasqualucci, Lea T. Grinberg, Paulo Mattos, Fernanda Tovar-Moll, Roberto Lent

**Affiliations:** 1 Institute of Biomedical Sciences, Federal University of Rio de Janeiro, Rio de Janeiro, RJ Brazil; 2 D’Or Institute for Research and Education (IDOR), Rio de Janeiro, RJ Brazil; 3 National Center for Structural Biology and Bioimaging (CENABIO), Rio de Janeiro, RJ Brazil; 4 Institute of Medical Sciences, Federal University of Rio de Janeiro, Macaé, RJ Brazil; 5 Department of Physiotherapy, Arcoverde Higher Education Authority (AESA), Arcoverde, PE Brazil; 6 Biobank for Aging Studies, University of São Paulo Medical School, São Paulo, SP Brazil; 7 Department of Medical Surgical Nursing, University of São Paulo School of Nursing, São Paulo, SP Brazil; 8 Division of Geriatrics, University of São Paulo Medical School, São Paulo, SP Brazil; 9 Department of Pathology, University of São Paulo Medical School, São Paulo, SP Brazil; 10 Department of Neurology, University of São Paulo Medical School, São Paulo, SP Brazil; 11 Department of Neuroscience and DLMP, Mayo Clinic, Jacksonville, FL USA

**Keywords:** Alzheimer’s disease, Cell number, Diffusion tensor imaging, DTI, Diffusivity, Medial temporal lobe

## Abstract

Volumetric and diffusion tensor imaging (DTI) alterations in medial temporal lobe (MTL) regions are consistently reported in Alzheimer's disease (AD). However, their biological basis remains poorly understood. We combined Magnetic Resonance Imaging (MRI) metrics with cellular quantification and neuropathological burden assessment to investigate microstructural substrates underlying MTL imaging alterations in human donors with known cognitive and neuropathological status. Absolute cell numbers were quantified in a cohort of 54 brain donors using the isotropic fractionator, and *postmortem* MRI was performed on a subset of 18 fixed brain hemispheres. To assess microstructural brain alterations associated with disease progression, in vivo volumetric and DTI metrics were analyzed in 39 cognitively unimpaired controls (CU), 31 individuals with mild cognitive impairment (MCI), and 14 patients with clinical AD dementia. In the *postmortem* cohort, MTL diffusivity metrics showed moderate negative correlations with total cell numbers. Mean, axial, and radial diffusivity positively correlated with Braak stage, whereas fractional anisotropy (FA) correlated negatively with CERAD score, linking diffusion alterations to AD-related pathology. In vivo MTL analyses revealed increased mean, axial, and radial diffusivity, and reduced FA in AD patients compared with both CU and MCI individuals. The MCI group exhibited intermediate alterations in diffusivity. Differences between in vivo and *postmortem* diffusion values were consistent with tissue fixation, temperature, and variations in acquisition protocols. Our findings suggest that diffusion MRI alterations in the MTL are associated with reduced cellularity and neuropathological burden, supporting the biological relevance of DTI metrics as markers of microstructural degeneration in AD.

**Supplementary Information:**

The online version contains supplementary material available at https://doi.org/10.1007/s00429-026-03223-4.

## Introduction

Magnetic resonance imaging (MRI) is a widely used tool to characterize structural changes in the brain associated with normal aging and neurodegenerative disorders. Among its modalities, Diffusion-Weighted Imaging (DWI) is highly sensitive to tissue microstructure by probing the displacement of water molecules within the biological tissue architecture (Basser and Jones 2002; Soares et al. 2013). It has been used in the study of white matter (WM) disorders and is increasingly applied to gray matter (GM) (Weston et al. 2015). The diffusion properties of the nervous tissue can be characterized using the diffusion tensor model, from which diffusion tensor imaging (DTI)-derived metrics, including Fractional Anisotropy (FA), Mean Diffusivity (MD), Axial Diffusivity (AxD), and Radial Diffusivity (RD), can be obtained. DTI parameters have been more extensively validated for WM (Alexander et al. 2007; Zhang et al. 2014), whereas the biological interpretation of diffusion abnormalities in GM remains less clearly defined.

In white matter, myelinated axons represent important elements that generate DTI signal through water diffusion restriction. High MD values have been associated with bimodal or diffusely distributed fiber orientations, as well as with low myelin density (Seehaus et al. 2015; van Veluw et al. 2019), low AxD has been associated with fiber injury (Soares et al. 2013), and FA has been found to correlate positively with the axonal number and density, myelination, and coherence of fiber orientation (Seehaus et al. 2015; Friedrich et al. 2020).

In the GM, the biological interpretation of DTI-derived metrics remains less well established due to the complex and relatively isotropic cytoarchitecture, in which myelinated axons represent only a small component compared to WM. Reveley et al. (2022) studied the GM of a marmoset brain, comparing ultra-high-resolution ex vivo diffusion MRI with co-registered Nissl and myelin histology. In contrast to WM, FA showed poor correspondence to the spatial distribution of myelin in GM. Better, FA was associated with unmyelinated tissue structures, typically vertically oriented, such as the large-caliber apical neurites of pyramidal neurons in infragranular layers (Reveley et al. 2022).

In humans, Goubran et al. (2015) combined in vivo MRI with quantitative histopathology to study the temporal neocortex of patients with focal epilepsy. The authors demonstrated that alterations of FA are predictive of neuronal integrity, particularly the density of large-caliber axons (specifically within layers III and V). They hypothesized that, in regions with complex fiber-crossings, such as the neocortex, this relationship could be explained by the selective loss of fibers from this type of neuron, resulting in a simpler fiber configuration that directly impacts anisotropy (Goubran et al. 2015). These results provide a biological basis for interpreting GM diffusion alterations as markers of dendritic and neuronal degeneration. Nevertheless, the histological substrates of diffusion MRI metrics in the human brain remain incompletely understood, particularly in neurodegenerative diseases such as Alzheimer’s disease.

*Postmortem* MRI enables the correlation of imaging findings with microscopic details. It offers improved image quality and a higher signal-to-noise ratio through longer acquisition times, the absence of motion and pulsation artifacts, and the use of contrast agents (Oguz et al. 2013; Shatil et al. 2018). The brain’s absolute cell number (cellularity) is a key measure of tissue microstructure, as many physiological and pathological processes in the nervous system are associated with changes in cellularity. Cell composition can be studied through *postmortem* histological processing using the isotropic fractionator, a technique that provides reliable estimates of absolute cell numbers in the brain (Herculano-Houzel and Lent 2005).

In this study, we focused on the human medial temporal lobe (MTL), a key region for learning and memory (Frankó et al. 2014), with significant differences in cellular composition according to sex and age (Oliveira-Pinto et al. 2016). MTL is one of the earliest affected regions in AD (Ravikumar et al. 2024), with individuals with dementia presenting with neuronal losses that can reach ~ 50% (Andrade-Moraes et al. 2013). Besides neuronal loss, AD is neuropathologically characterized by the accumulation of extracellular amyloid-β plaques and intracellular neurofibrillary tangles composed of hyperphosphorylated tau (Serrano-Pozo et al. 2011).

Although volumetric and diffusion MRI alterations in the MTL are widely reported in AD, the underlying microstructural substrates remain poorly understood. This study addresses a fundamental question: What are the specific cellular and neuropathological substrates that drive volumetric and diffusion-tensor imaging alterations in the human medial temporal lobe? To address this question, we hypothesized that cellular composition and neuropathological alterations are associated with changes in diffusivity metrics. Finally, we explored the microstructural MRI signatures in vivo to determine if there was a profile across the clinical AD continuum, from cognitively unimpaired to Mild Cognitive Impairment (MCI) and clinical AD.

To test our hypothesis, we investigated *postmortem* volumetric and DTI alterations in the MTL of subjects with dementia of different etiologies, mainly AD. The same material was then processed to establish their relationships with cell numbers and AD-related neuropathological burden. Mapping imaging metrics together with absolute cellularity is impossible in living subjects. Therefore, our *postmortem* cohort serves as the mechanistic foundation of this study. By combining high-resolution *postmortem* MRI with neuropathological staging and quantitative cellular analysis using the isotropic fractionator in the same brain tissue, we establish direct, biologically validated links between imaging signals and the underlying cellular composition.

The translational value of *postmortem* MRI, however, depends on its integration with in vivo imaging and quantitative microstructural data. While *postmortem* imaging provides biological validation, it is subject to fixation, temperature, and death-related artifacts. Thus, validating these *postmortem*-derived imaging signatures in an independent in vivo cohort is essential to demonstrate their practical utility as clinical biomarkers of disease progression. Along these lines, this study aimed to clarify the cellular and neuropathological substrates underlying volumetric and diffusion MRI alterations in dementia, with particular emphasis on AD, and subsequently evaluate the profile of these MRI metrics in vivo across the clinical AD continuum.

## Materials and methods

### Experimental design

This study employed two complementary approaches: *postmortem* and in vivo. In the *postmortem* approach, volumetric and DTI metrics of the MTL were obtained from fixed brain hemispheres of individuals with known cognitive status and complete histopathological diagnosis, confirming the presence or absence of AD neuropathological findings and other etiologies of dementia. Imaging measures were then correlated with absolute cell number and AD-related pathological burden. In the in vivo approach, the same MRI metrics were analyzed in a different cohort of patients with clinical diagnoses of AD, MCI, and no cognitive decline, to assess their structural features. The *postmortem* study was designed to establish the biological correlates of MRI-derived metrics, whereas the in vivo study aimed to evaluate the clinical expression of these imaging signatures.

### Postmortem study

#### Sample and cognitive assessment

*Postmortem* procedures were approved by institutional ethics committees (Nº 588/10, Federal University of Rio de Janeiro; Nº 458.272/2013, University of São Paulo Medical School; Nº 032/2015 - Nº 25000.050648/2014-61, Brazilian National Committee of Ethics in Research, CONEP). In all cases, written informed consent for brain donation was obtained from the deceased’s next of kin. Cognitive status was determined using the *Clinical Dementia Rating* (CDR) scale (Morris 1993). This tool was previously adapted by the Biobank for Aging Studies for use in *postmortem* studies (Ferretti et al. 2010; Ferretti-Rebustini et al. 2021), based on informant interviews conducted by trained personnel. Information was considered reliable if the informant had contact with the subject at least once a week during the last six months before death. Inclusion criteria comprised age 60 years or older and cognitive status (CDR = 0, cognitively unimpaired; CDR = 3, advanced dementia).

Table 1 summarizes the demographic characteristics of the donors. For exclusion criteria, see Supplementary Material S2. The sample consisted of 54 subjects distributed across five groups based on cognitive status and neuropathological assessment (NP). Specifically, the sample comprised: 33 Controls (CDR = 0 and NP classified as normal), 7 cases with Asymptomatic AD (AsymAD, CDR = 0 and AD neuropathology), 2 cases with other asymptomatic dementias (Other AsymDem, CDR = 0 and neuropathological findings consistent with other dementias, Lewy bodies disease and Progressive Supranuclear Palsy), 10 cases with AD dementia (CDR = 3 and NP consistent with AD) and 2 cases with Other dementias (CDR = 3 and NP consistent with other causes of dementia).

#### Brain dissection and hemisphere allocation

**Fig. 1:**
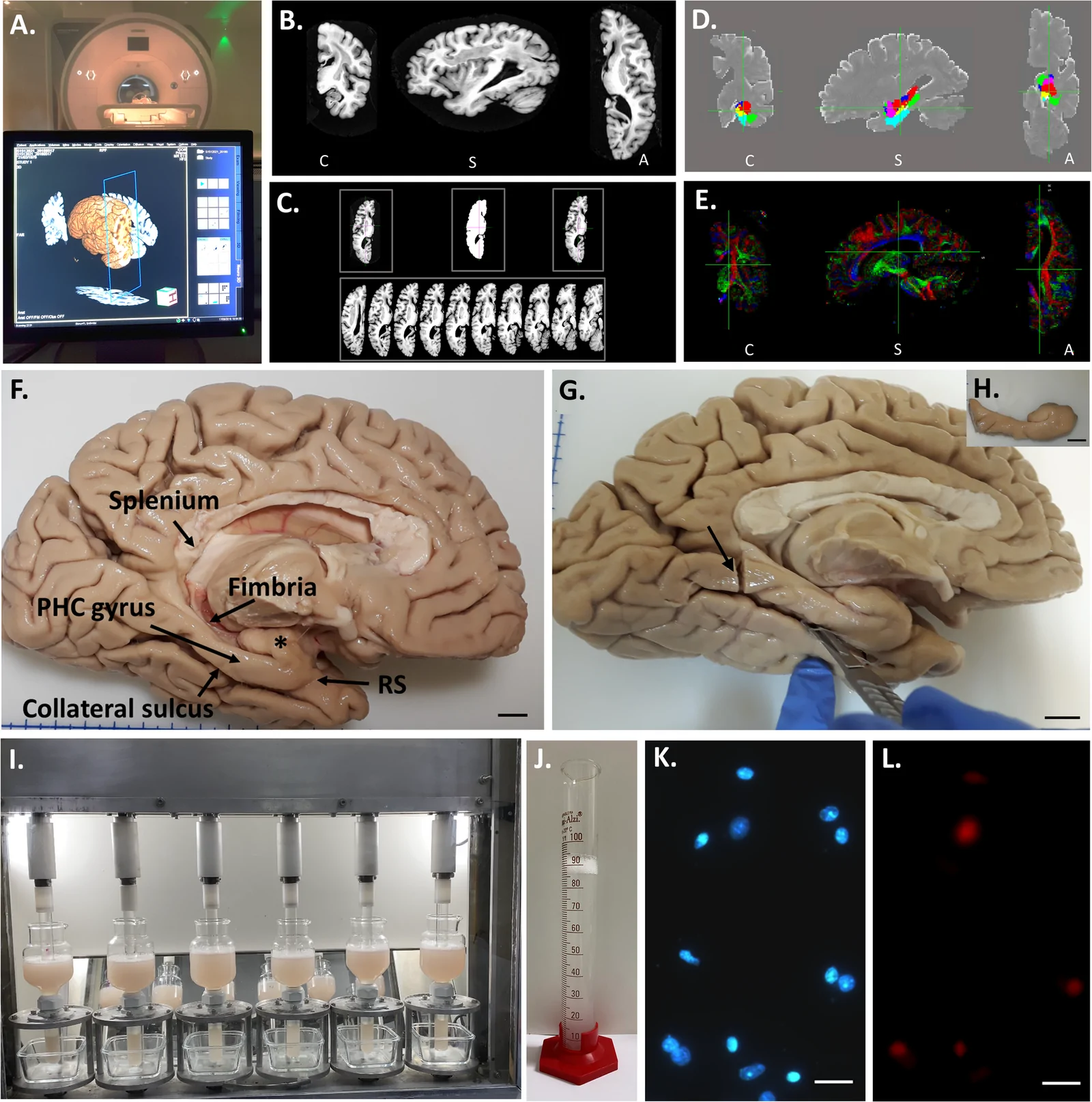
Left Medial Temporal Lobe processing. **A** MRI acquisition in a 3T Siemens Magnetom Prisma. **B** Structural MRI. **C** Structural MRI processing. **D** Diffusion MRI, MD map, ROI: Medial Temporal Lobe. **E** Diffusion MRI, Colored FA map. **F** Medial surface of the left hemisphere, showing the identification of landmarks, RS: Rhinal sulcus; *: Uncus, PHC: Parahippocampal cortex. **G** MTL dissection (arrow shows posterior cutting plane at the splenium caudal pole). **H** Dissected MTL. **I** Semi-automated chemomechanical dissociation. **J** Isotropic suspension of nuclei. **K** Cell nuclei stained with DAPI. **L** NeuN-positive nuclei. *Scale bar*: 1 cm (**F**, **G**, **H**), 20 μm (**K**, **L**)

The brains were dissected apart from the spinal cord by a transverse section through the inferior limit of the brainstem at the *foramen magnum* level. They were then split at the sagittal plane to separate the two hemispheres. The *postmortem* interval (PMI) was less than 26 h in all cases. The right hemisphere was allocated for histopathological analysis, and the left hemisphere for MRI acquisition and cell quantification (Fig. 1).

#### Neuropathological assessment

The right hemisphere was fixed by immersion in 4% buffered paraformaldehyde solution. After fixation, samples were collected for histopathological analysis (Supplementary Material S2). Each sample was paraffin-embedded and cut into 5 μm sections. Each section was mounted on glass slides, deparaffinized, rehydrated, and stained with hematoxylin and eosin for general histopathological evaluation. Immunohistochemistry was performed for the following antibodies: Phospho-Tau (Ser202, Thr205) monoclonal antibody (AT8, 1:400), Anti-β-Amyloid (17–24) monoclonal antibody (4G8, 1:10000), Purified anti-TDP43 Phospho (Ser409/410) monoclonal antibody (1D3/TDP-43, 1:500) and Purified anti-α-Synuclein Phospho (Ser129) monoclonal antibody (P-syn/81A, 1:500). Neuropathological diagnosis was based on internationally accepted criteria: Alzheimer’s Disease (Consortium to Establish a Registry for Alzheimer’s Disease (CERAD): Mirra et al. 1991; and Braak: Braak and Braak 1991), Frontotemporal Lobar Degeneration (FTLD) (Cairns et al. 2007), Limbic-predominant Aged-related TDP-43 Encephalopathy (LATE) (Nelson et al. 2019), Lewy Body Disease (Braak et al. 2003), and hippocampal sclerosis. AD was diagnosed when the Braak staging (Braak and Braak 1991) was equal to or greater than III, and CERAD score for neuritic plaques was moderate or severe (Mirra et al. 1991).

#### Postmortem MRI acquisitions

A subsample of these cases was imaged (*n* = 18: Table 2 summarizes group data, and individual characteristics are provided in Table S1). The left hemisphere was fixed in 2% Paraformaldehyde in Phosphate Buffered Saline (PBS) for 36–40 h at 4 °C and then maintained in PBS at 4 °C. The MRI protocol was standardized for a Siemens Magnetom Prisma 3T (Siemens Healthcare, Erlangen, Germany) (Fig. 1A), using a 64-channel head coil. 

MRI data were acquired in the coronal plane (Fig. 1B, C) and included the following sequences: (i) a T1-weighted MPRAGE sequence (TR/TE = 2,300/2.27 ms, FOV = 192 × 192 mm, matrix = 192 × 192, voxel size = 1.0 × 1.0 × 1.0 mm³); (ii) Multi–echo acquisition with flip angles of 5°, 10°, 15°, 20°, 25°, and 30° (TR = 36 ms, matrix = 192 × 192, FOV = 192 × 192 mm, voxel size = 1.0 × 1.0 × 1.0 mm³; total acquisition time = 12 min per flip angle), with the following Echo Times: TE_1_: 2.46 ms, TE_2_: 4.92 ms, TE_3_: 7.38 ms, TE_4_: 9.84 ms, TE_5_: 12.30 ms, TE_6_: 14.76 ms, TE_7_: 17.22 ms, TE_8_: 19.68 ms, TE_9_: 22.14 ms, TE_10_: 24.60 ms, TE_11_: 27.06 ms, TE_12_: 29.52 ms; and (iii) diffusion-weighted imaging acquired using a fast spin echo sequence with diffusion gradients applied in 64 directions (TR/TE = 9,300/93 ms, FOV = 192 × 192 mm, voxel size = 1.5 × 1.5 × 1.5 mm³, 50 slices, b = 3000 s/mm², acquisition time = 81 min). More details of *Postmortem* MRI acquisitions in the Supplementary Material S3.

#### **Postmortem image processing and analysis**

Briefly, images were converted to NIFTI format and processed using MRtrix3 (v3.0.4; https://www.mrtrix.org/) and FSL (v6.0.4; https://fsl.fmrib.ox.ac.uk/fsl/fslwiki/) (Jenkinson et al. 2012). The quality, number of volumes, and orientation in the x, y, and z axes were checked for each image. Similar to in vivo image processing, all diffusion images were first denoised (dwidenoise, MRtrix 3.0.3; Veraart et al. 2016) and corrected for Gibbs ringing artifacts (mrdegibbs, MRtrix 3.0.3; Kellner et al. 2016). Then, the diffusion images were corrected for motion and eddy geometric distortions induced by diffusion gradients (dwifslpreproc, MRTRIX 3.0.3 and FSL v6.0.4; Andersson et al. 2003), followed by bias field correction and image normalization (dwibiascorrect, MRtrix 3.0.3; Tustison et al. 2010). A binary mask was created from predefined thresholds to remove the unnecessary appearance of non-brain areas, such as the plastic container and PBS.

After the tensor estimation, a visual examination of the tensor orientation was performed in specific regions (corpus callosum, cingulate gyrus, and pyramidal tract). No errors were detected in the tensor orientation, so the gradient direction table was maintained. Diffusion tensors were calculated at each voxel, and FA, MD, RD, and AxD maps (Fig. 1E) were derived using the DTIFIT tool (FSL). Due to single-hemisphere acquisition, it was necessary to adapt and modify pipelines developed for analyzing in vivo MRI, so the Montreal Neurological Institute (MNI) template was split along the latero-lateral axis. Subsequently, each T1 image was linearly registered to the B0. MTL region was defined using standard atlases (Harvard–Oxford, Jülich, and Johns Hopkins University, JHU), including hippocampus, amygdala, entorhinal cortex, parahippocampal gyrus, and related subregions. ROIs (Fig. 1D) matched those used in the in vivo analysis and were manually corrected when necessary. Following the in vivo analyses (see below), *fslstats* was used to calculate the mean values of each tensor metric in MTL ROI.

#### Cell quantification

A subset of the cellularity data used in the present study has been previously reported in other publications addressing different research questions. Previous studies have shown that MTL cellular composition varies according to sex, age, and the presence of AD (Andrade-Moraes et al. 2013; Oliveira-Pinto et al. 2016), with no significant variations associated with educational level (de Morais et al. 2023). Following MRI, the MTL was dissected from the left hemisphere using standardized anatomical landmarks (Fig. 1F-H) (For more details of the dissection, see the Supplementary Material S4). Total cell and neuronal numbers were quantified using the isotropic fractionation method, as previously described (Herculano-Houzel and Lent 2005; Azevedo et al. 2009, 2013). Briefly, tissue samples were chemomechanically dissociated (Fig. 1I) in sodium citrate and Triton X-100, yielding a homogeneous suspension of nuclei (Fig. 1J). Total cell number was estimated by counting DAPI-stained nuclei (Fig. 1K) in a hemocytometer, with coefficients of variation < 15%. Neuronal proportion was determined by immunolabeling with NeuN (Fig. 1L), and absolute numbers of neurons and non-neuronal cells were calculated accordingly (For more details of cell quantification, see the Supplementary Material S4).

### In vivo study

#### Participants

This part of the study was approved by the Ethics Committee of the D’Or Institute for Research and Education (IDOR; Nº 226/2011). All participants signed a written informed consent for imaging acquisition. The cohort was previously described (Drummond et al. 2019) and included 39 cognitively unimpaired controls (CU), 31 individuals with mild neurocognitive disorder (Mild cognitive impairment, MCI), and 14 patients with major neurocognitive disorder due to Alzheimer’s disease (AD). The inclusion criteria comprised subjects aged between 60 and 85 years, with Brazilian Portuguese as their mother language, formal education higher than or equal to eight years, completed language and neuropsychological batteries, and MRI evaluation. The diagnoses were made using the Diagnostic and Statistical Manual of Mental Disorders (DSM-5) criteria (American Psychiatric Association 2013). Exclusion criteria included frontotemporal dementia, primary progressive aphasia, dementia with Lewy bodies, advanced cerebrovascular disease, non-amnestic MCI and any major neuropsychiatric disorder report (schizophrenia or other psychotic disorders, bipolar disorder, epilepsy, alcohol or substance abuse, current severe depressive disorder or severe head injury).

#### MRI acquisitions

Images were acquired in a 3T Philips Achieva MRI scanner (Philips Healthcare, Best, the Netherlands). The protocol included: (i) a high-resolution isotropic 3D T1-weighted sequence (TR/TE = 7.2/3.4 ms, Field of view (FOV) = 240 × 240 mm, matrix = 240 × 240, 170 slices, voxel size = 1.0 × 1.0 × 1.0 mm³); and (ii) Diffusion-Weighted Images (DWI): a spin-echo echo-planar sequence in the axial plane; TR/TE = 5,582/65 ms, FOV = 240 × 240 mm, matrix = 96 × 95, 60 slices, voxel size = 2.5 × 2.5 × 2.5 mm³, 32 non-collinear diffusion directions, and a b-value of 1000 s/mm².

#### Image processing and analysis

Structural MRI data were processed using FreeSurfer (version 6.0.0, https://surfer.nmr.mgh.harvard.edu) (Fischl 2012). The pipeline included intensity normalization, skull stripping, and segmentation. The same MTL ROI definition was used for the in vivo analysis and was manually corrected when necessary. The cortical thickness and volumetric measures were extracted for each ROI.

In vivo diffusion images were processed using an in-house pipeline similar to that described for the *postmortem* data, using open-source tools (MRtrix3 and FSL). To ensure the quality of the acquired data for each subject, all diffusion images were visually inspected for artifacts. Processing steps included denoising, Gibbs ringing removal, motion and eddy-current, bias field correction, and image normalization. A brain mask image was generated (Dhollander et al. 2016) to remove non-brain tissue from diffusion-weighted images. After this, the FA, MD, AxD, and RD maps were generated by fitting each DWI dataset to the diffusion tensor model. ROIs were registered to the diffusion space, and mean values of FA, MD, AxD, and RD were extracted from each ROI (see the *Postmortem* image processing and analysis section for a complete description of the tools).

### Statistical analyses

Statistical analyses were performed using GraphPad Prism 8 (GraphPad Software, San Diego, CA, USA) and Jamovi statistical software (version 2.7.15.0; The Jamovi project). Data distribution was assessed for normality prior to statistical testing. Individual outliers were identified and removed prior to analysis using the interquartile range (IQR) method, defined as values lying below Q1 − 1.5×IQR or above Q3 + 1.5×IQR. Only the outlying observation for the corresponding MRI measure was excluded from that specific analysis; these exclusions did not alter the overall pattern of results. The number of excluded observations for each MRI measure is summarized in Supplementary Material S5 (Table S2). Differences in categorical variables among groups were assessed using the chi-square (χ²) or Fisher’s exact test, according to sample size. Age was compared between groups using one-way ANOVA or Kruskal–Wallis test, depending on data distribution.

For the *postmortem* analyses, imaging parameters were compared between groups using a general linear model including sex, age, and co-pathologies as covariates. Each co-pathology was included as a separate binary covariate (present/absent). Given the limited sample size, correlations between cell numbers and histological variables were assessed using Spearman’s rank correlation coefficient. Due to the correlation analyses being restricted to a priori hypotheses, nominal p-values are reported together with effect sizes (Spearman’s ρ) and 95% confidence intervals. No formal correction for multiple comparisons was applied to the correlation analyses.

For the in vivo analyses, MRI metrics were compared across groups using analysis of covariance (ANCOVA), with age and sex included as covariates. Post hoc pairwise comparisons of estimated marginal means were performed using Tukey’s multiple comparison correction. Comparisons between in vivo and *postmortem* diffusion metrics were performed using unpaired t-tests. Statistical significance was set at *p* < 0.05.

#### Cellularity prediction model

To explore whether *postmortem* MRI metrics could predict total cellular count, we fitted two regression-based models: a standard multiple linear regression and a ridge regression model with L2 regularization. A detailed description of the modeling procedures and full results is provided in Supplementary Material S6.

## Results

### Postmortem

#### Demographic characteristics

The *postmortem* sample comprised 54 subjects, categorized into five groups based on cognitive status and NP assessment (for details, see Materials and Methods). Table 1 summarizes the demographic characteristics of the study participants. Age differed significantly among groups (Kruskal–Wallis test, *p* = 0.010). Post-hoc pairwise comparisons revealed differences between the control group and the AD dementia group (*p* = 0.022). The highest mean ages were observed in the AD dementia group (82.80 ± 7.77 years), whereas the control group showed a lower mean age (72.33 ± 9.33 years). Sex distribution also differed significantly between groups (Fisher’s exact test, *p* = 0.002).

**Table 1:** Demographic characteristics of brain donors

	Control	AsymAD	Other AsymDem	AD dementia	Other dementias	*p*-value
	CDR = 0	CDR = 0	CDR = 0	CDR = 3	CDR = 3	
n	33	7	2	10	2	
Age, mean (SD)	72.33 (9.33)	81.71 (3.15)	80.50 (21.92)	82.80 (7.77)	79.50 (3.54)	0.010*
Sex: F/M	12/21	6/1	0/2	9/1	1/1	0.002**

*AD* Alzheimer’s disease, *AsymAD* Asymptomatic Alzheimer’s disease, *CDR* Clinical Dementia Rating, *F* Female, *M* Male, *Other AsymDem* other asymptomatic dementias, *SD* Standard Deviation

#### Cellular composition of the medial temporal lobe

**Fig. 2:**
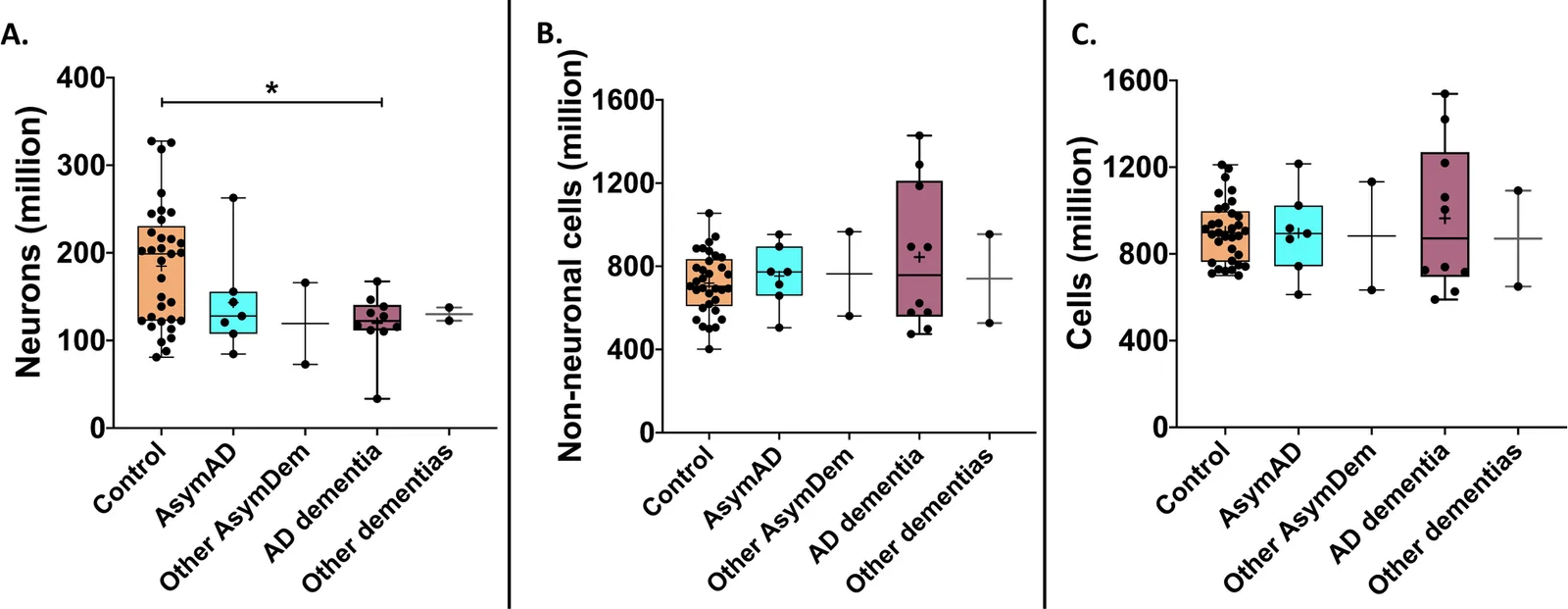
Cellular composition in the left medial temporal lobe across diagnostic groups in the *postmortem* sample. **A** Total number of neurons. **B** Total number of non-neuronal cells. **C** Total number of cells. Boxes represent the median and interquartile range, crosses indicate the mean, and dots represent individual subjects. *p < 0.05

To determine whether neuronal and non-neuronal cellular composition differed according to cognitive and neuropathological status, we compared the absolute numbers of neurons, non-neuronal cells, and total cells across the diagnostic groups. A significant overall effect of diagnostic group on the total number of neurons was detected in the medial temporal lobe (F (4,49) = 2.79, *p* = 0.036, partial η² = 0.186). Post-hoc pairwise comparisons using Tukey correction showed that AD patients had a significantly lower number of neurons compared to controls (mean difference = 64.94, CI [1.47, 128.40], *p* = 0.043). No other pairwise comparison between diagnostic groups reached statistical significance. In contrast to neuronal counts, no significant group differences for non-neuronal cells or total cell numbers (*p* = 0.592 and *p* = 0.933, respectively) (Fig. 2A-C).

 These findings indicate that neuronal loss, rather than changes in non-neuronal or total cellularity, is the main cellular alteration distinguishing AD dementia from cognitively normal controls in the MTL.

 A subsample of these cases was imaged (*n* = 18, Table 2 summarizes group data; individual characteristics are provided in Table S1), including 4 controls, 3 AsymAD, 2 cases with Other AsymDem, 7 cases with AD dementia, and 2 cases with other causes of dementia. Age (Kruskal–Wallis test, *p* = 0.746) and sex distribution (Fisher’s exact test, *p* = 0.281) did not differ significantly among groups.

**Table 2:** Postmortem MRI-imaged brain sample

	Control	AsymAD	Other AsymDem	AD dementia	Other dementias	*p*-value
	CDR = 0	CDR = 0	CDR = 0	CDR = 3	CDR = 3	
n	4	3	2	7	2	
Age Med (IQR)	80.00 (10.00)	85.00 (4.00)	80.5 (15.5)	88.00 (8.00)	79.50 (2.50)	0.746
Sex: F/M	3/1	2/1	0/2	6/1	1/1	0.281

*AD* Alzheimer’s disease, *AsymAD* Asymptomatic Alzheimer’s disease, *CDR* Clinical Dementia Rating, *F* Female, *IQR* Interquartile Range, *M* Male, *Med* Median, *Other AsymDem* other asymptomatic dementias

Although our initial aim was to investigate Alzheimer’s disease, the final *postmortem* cohort proved to be neuropathologically heterogeneous following the comprehensive neuropathological assessment. Rather than representing a limitation solely, this heterogeneity ultimately proved advantageous, as it increased the variability in neuropathological burden and tissue cellularity, providing a broader biological spectrum to examine continuous associations between MRI metrics and their underlying histopathological substrates. Because our primary objective was to identify the cellular correlates of MRI-derived measures rather than distinguish dementia subtypes, this broader pathological spectrum strengthened the biological interpretation of the observed imaging findings.

#### Structural and diffusion metrics

**Fig. 3:**
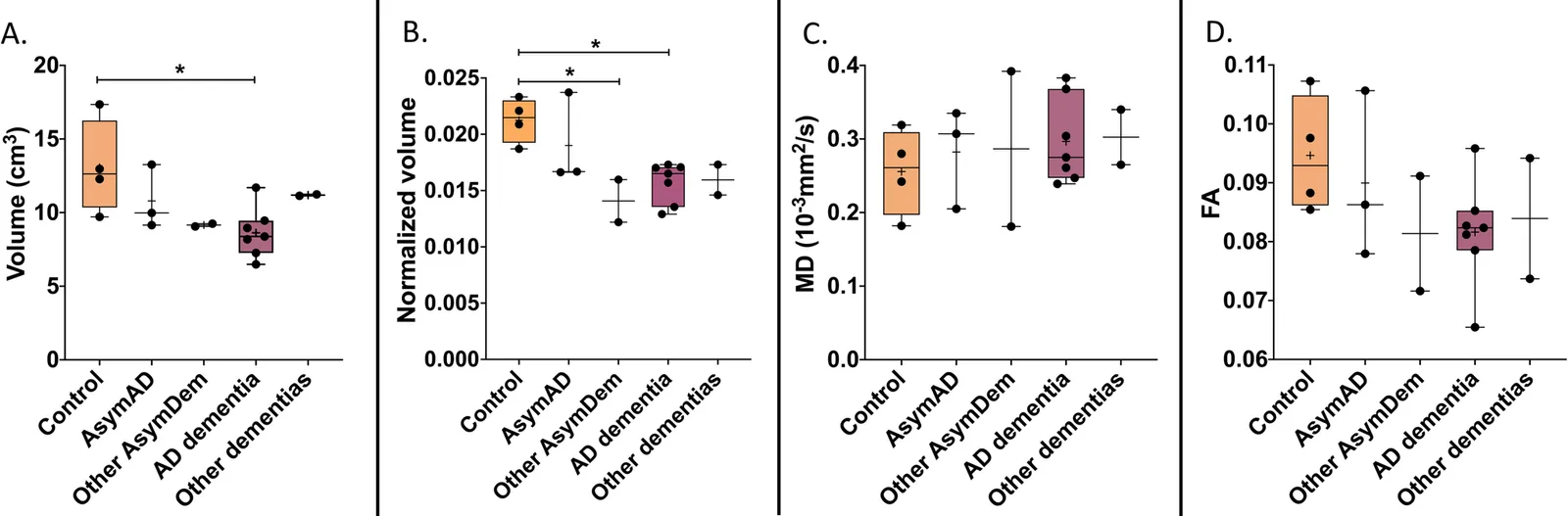
Group differences in *postmortem* MRI metrics of the medial temporal lobe. **A** Volume. **B** Normalized volume **C** Mean diffusivity (MD). **D** Fractional anisotropy (FA). Boxes represent the median and interquartile range, crosses indicate the mean, and dots represent individual subjects. *p < 0.05

To determine whether *postmortem* MRI metrics capture structural and microstructural differences associated with disease, we compared MTL volumetric and diffusion measures across diagnostic groups. MTL volume differed across groups (F (4,13) = 3.18, *p* = 0.049, partial η² = 0.495), with lower values observed in the AD dementia group compared with controls (mean difference = 4.45, CI [0.33, 8.57], *p* = 0.032) (Fig. 3A). After normalization (correcting for hemisphere volume), the same pattern was observed (F (4,13) = 4.89, *p* = 0.013, partial η² = 0.601), and the control group exhibited higher normalized volumes compared with Other AsymDem (*p* = 0.030) and AD dementia (*p* = 0.020) (Fig. 3B).

MD values tended to be higher in the AD dementia group compared with controls, although no statistically significant differences were found among groups (*p* = 0.903) (Fig. 3C). Similar results were observed for AxD and RD (*p* = 0.920 and *p* = 0.898, respectively ). FA values showed a tendency toward lower FA in the other groups compared to the control, but without clear significant differences (*p* = 0.402) (Fig. 3D).

 Given the limited sample size within each diagnostic category, we additionally pooled all non-control groups and compared them with the control group. This complementary analysis did not reveal significant differences in MD (*p* = 0.320) and did not reach statistical significance in FA (*p* = 0.083). Together, these results indicate that volumetric MRI detects structural differences between diagnostic groups, whereas diffusion metrics did not show significant group-level differences in this *postmortem* cohort. Subsequent correlation analyses were conducted to determine whether diffusion measures nevertheless reflect underlying cellular composition and neuropathological burden independent of overt group differences.

#### Associations between diffusion MRI metrics and cellular composition in dementia

To test our first hypothesis, that absolute cell composition drives tissue diffusion changes, we performed correlation analyses between *postmortem* MTL DTI metrics and absolute cell numbers quantified via the isotropic fractionator. This analysis examined whether alterations in water diffusion can reflect underlying cellularity in this region.

**Fig. 4:**
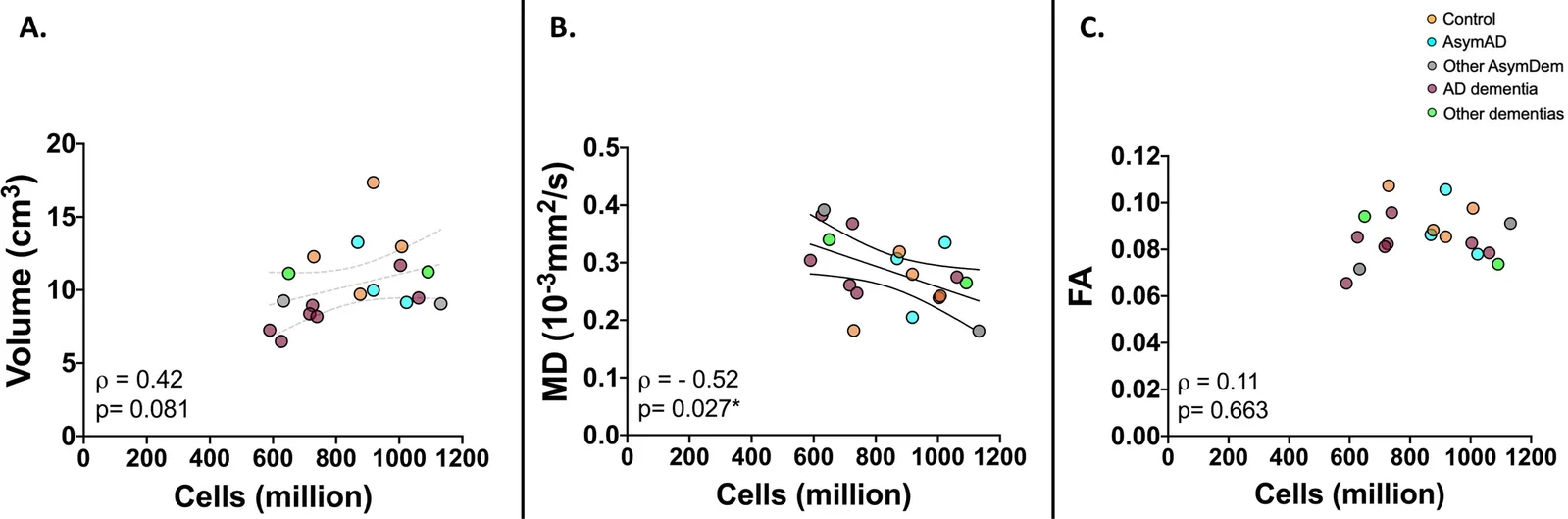
Associations between diffusion MRI metrics and cellularity. **A** Volume and cellularity. **B** MD and cellularity. **C** FA and cellularity. Each dot represents one subject, with colors indicating diagnostic groups: Control subjects, asymptomatic Alzheimer’s disease (AsymAD), Other asymptomatic dementias (Other AsymDem), Alzheimer’s disease dementia (AD dementia), and other dementias. Spearman correlation coefficients (ρ) and *p*-values are displayed in each panel. The lines represent the linear regression fit with 95% confidence intervals

**Fig. 5:**
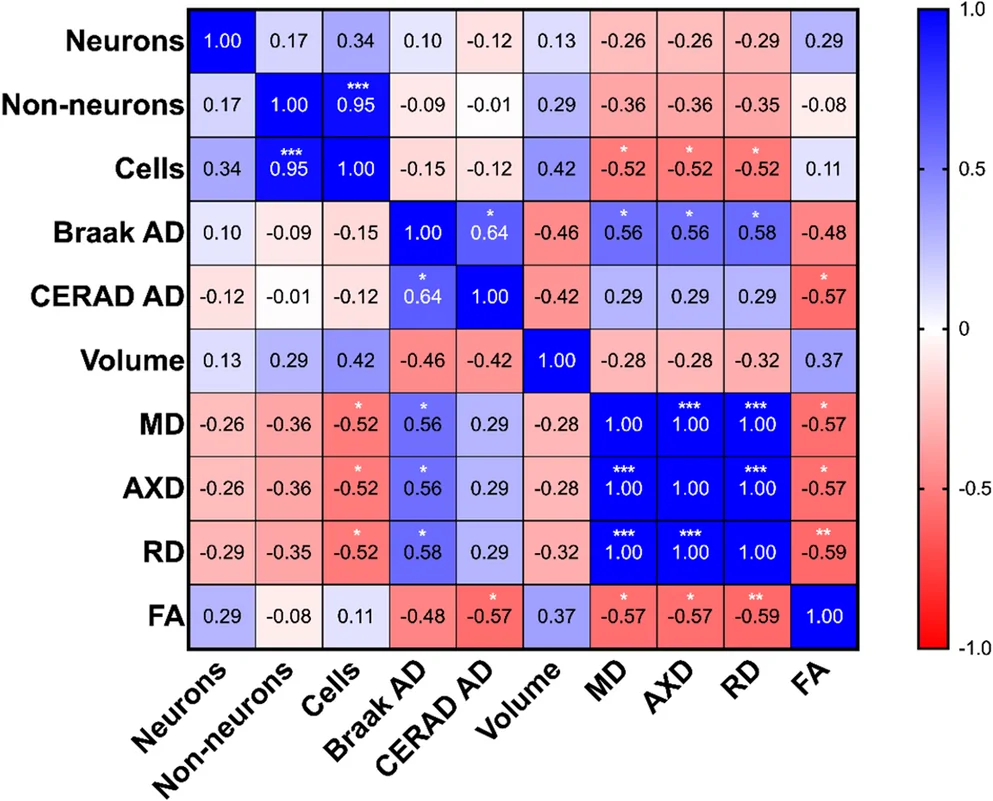
Correlation matrix between cellularity, histopathology, and MRI parameters. The heatmap displays pairwise Spearman correlation coefficients between cell numbers, histopathological findings, and MRI-derived parameters. The color scale indicates the strength and direction of the correlation (blue = positive; red = negative). Statistically significant correlations are denoted as follows: *p < 0.05, **p < 0.01. ***p < 0.001

A positive trend was observed between the total number of cells and MTL volume; however, this association did not reach statistical significance (Spearman’s ρ = 0.42, CI [-0.07, 0.75], *p* = 0.081) (Fig. 4A). Importantly, despite the absence of group-level differences among groups, diffusion metrics showed significant negative correlation with cellular counts. MD showed a significant negative correlation with cellularity (Spearman’s ρ = -0.52, CI [-0.80, -0.05], *p* = 0.027) (Fig. 4B). Similar results were obtained for AxD (Spearman’s ρ = -0.52, CI [-0.80, -0.05], *p* = 0.027), and RD (Spearman’s ρ = -0.52, CI [-0.80, -0.10], *p* = 0.025) (Fig. 5). In contrast, FA showed no correlation with the number of cells (Spearman’s ρ = 0.11, CI [-0.39, 0.56], *p* = 0.663) (Fig. 4C).

Finally, to investigate whether MRI-derived measures could predict underlying cellular composition, we constructed a multiple linear regression model using MRI metrics as predictors of total cell number. However, the model was not robust, suggesting that MRI metrics alone were insufficient for estimating total cellularity accurately (Supplementary Material S6). These results suggest that although diffusion MRI metrics are associated with cellularity, they do not provide sufficient information to accurately predict absolute cell numbers, indicating that additional biological factors contribute to the observed MRI signal.

#### Associations between diffusion-weighted imaging parameters and the neuropathological burden in AD

To address our second hypothesis, we tested the association between MTL diffusion metrics and neuropathological findings, specifically tau and neuritic plaques staining, within controls and subjects with confirmed neuropathological diagnosis of AD. MRI parameters were correlated with the histopathological burden of Alzheimer’s disease, as assessed by Braak and CERAD stages. MD (Spearman’s ρ = 0.56, CI [0.03, 0.85], *p* = 0.039), AxD (Spearman’s ρ = 0.56, CI [0.03, 0.85], *p* = 0.039), and RD (Spearman’s ρ = 0.58, CI [0.05, 0.85], *p* = 0.032) demonstrated positive, moderate, and statistically significant correlations with Braak stage. In contrast, FA showed a negative and statistically significant correlation with CERAD AD (Spearman’s ρ = -0.57, CI [-0.85, -0.04], *p* = 0.035) (Fig. 5). As expected, strong positive correlations were observed between related histological measures, such as the number of non-neuronal cells and total cell counts (Spearman’s ρ = 0.95, CI [0.87, 0.98], *p* = 1.56 × 10⁻⁹). Similarly, diffusion parameters were significantly intercorrelated, reflecting the interdependence of these metrics (*p* < 0.05).

Although moderate and based on a small sample, these results suggest that, in addition to cellular composition, neuropathological burden is associated with diffusion changes in the MTL, with greater tau staging relating mainly to higher diffusivity metrics (MD, AxD, RD) and greater neuritic plaque burden to lower FA.

### In vivo

#### In vivo morphometric alterations of the medial temporal lobe in AD

To characterize the pattern of structural changes associated with disease progression and establish the morphometric alterations underlying subsequent diffusion analyses, we first evaluated regional morphometric measures of the medial temporal lobe.

Table 3 summarizes the demographic characteristics of the in vivo sample. The CU, MCI, and AD groups did not differ significantly in sex distribution (χ² test, *p* = 0.528). A significant group effect was observed for age (one-way ANOVA, *p* = 0.049), with the AD group showing a higher mean age. All the subsequent analyses were adjusted for age and sex.

**Table 3:** Demographic characteristics of the in vivo sample

*n*	CU	MCI	AD	*p*-value
	39	31	14	
Age mean (SD)	71.54 (4.24)	72.42 (4.86)	75.43 (6.99)	0.049*
Sex: F/M	25/14	16/15	9/5	0.528

* AD* Alzheimer’s disease, *CU* Cognitively unimpaired individuals, *F* Female, *M* Male, *MCI* Mild Cognitive Impairment, *SD* Standard Deviation

**Fig. 6:**
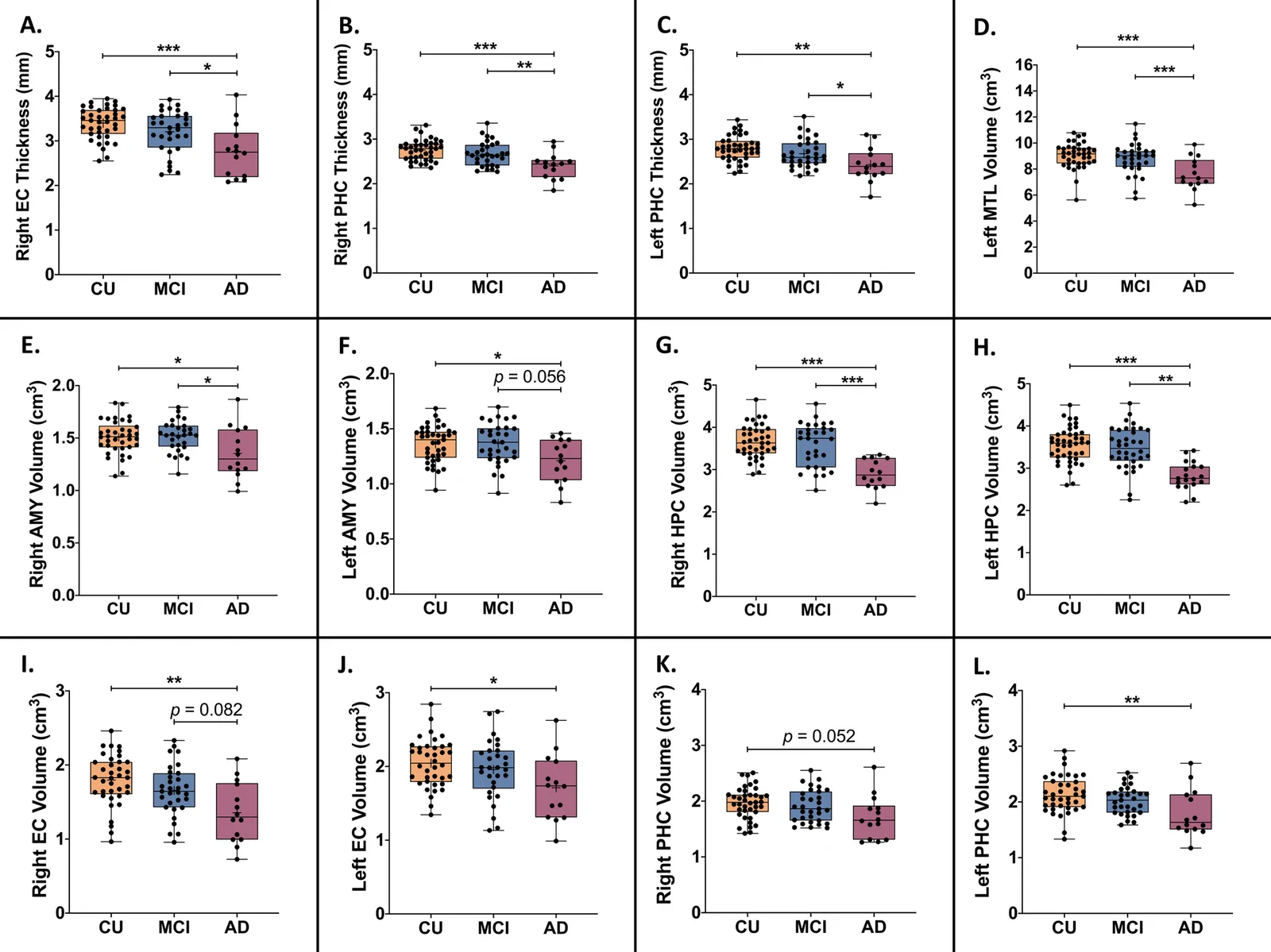
In vivo MRI morphometric differences in the left medial temporal lobe across diagnostic groups. **A** Right entorhinal cortex thickness. **B** Right parahippocampal cortex thickness. **C** Left parahippocampal cortex thickness. **D** Left MTL volume. **E** Right amygdala volume. **F** Left amygdala volume. **G** Right hippocampal volume. **H** Left hippocampal volume. **I** Right entorhinal cortex volume. **J** Left entorhinal cortex volume. **K** Right parahippocampal cortex volume. **C** Left parahippocampal cortex volume. *AD* Alzheimer’s Disease, *AMY* Amygdala, *CU* Cognitively unimpaired individuals, *EC* Entorhinal cortex, *HPC* Hippocampus, *MCI* Mild cognitive impairment, *PHC* Parahippocampal cortex. Boxes represent the median and interquartile range, crosses indicate the mean, dots represent individual subjects. Statistical significance was assessed using ANCOVA, followed by Tukey post hoc comparisons. Statistical significance is indicated as *p < 0.05, **p < 0.01, ***p < 0.001

At the regional level, medial temporal lobe structures showed statistically significant group differences. Cortical thickness of the right entorhinal cortex (Fig. 6A) and bilateral parahippocampal cortex (Fig. 6B, C), as well as volumes of the right amygdala and bilateral hippocampi (Fig. 6E, G, H), were significantly reduced in AD patients compared with both the CU and MCI group. Additional differences between CU and AD were observed for the volume of the bilateral entorhinal cortices (Fig. 6I, J) and of the left parahippocampal cortex (Fig. 6L).

Some comparisons showed trend-level effects. A trend was observed between CU and AD for right parahippocampal volume (*p* = 0.052; Fig. 6K) and between MCI and AD for right entorhinal volume (*p* = 0.082; Fig. 6I). No significant differences were found between CU and MCI for any of these measures.

A one-way ANCOVA revealed a significant main effect of diagnostic group on left MTL volume (F (2, 75) = 13.09, *p* < 0.001, partial η² = 0.26). Post hoc comparisons of estimated marginal means with Tukey correction showed that the AD had lower MTL volume compared with both the CU group (*p* < 0.001, Cohen’s d (*d*) =-1.658, CI [-2.362, -0.954]) and the MCI group (*p* < 0.001, *d* = -1.364, CI [-2.067, -0.660]), whereas no significant difference was observed between CU and MCI (*p* = 0.472, *d* = 0.294, CI [-0.207, 0.796]) (Fig. 6D).

Overall, these findings showed progressive medial temporal lobe atrophy across the Alzheimer’s disease continuum, with the most pronounced structural alterations observed in patients with AD. In contrast, morphometric changes in MCI compared to controls were not detected at this level.

#### In vivo diffusion alterations of the medial temporal lobe in AD

To test our third hypothesis that the cellular and pathological substrates, validated *postmortem*, translate into reliable clinical markers, we analyzed in vivo DTI metrics.

**Fig. 7:**
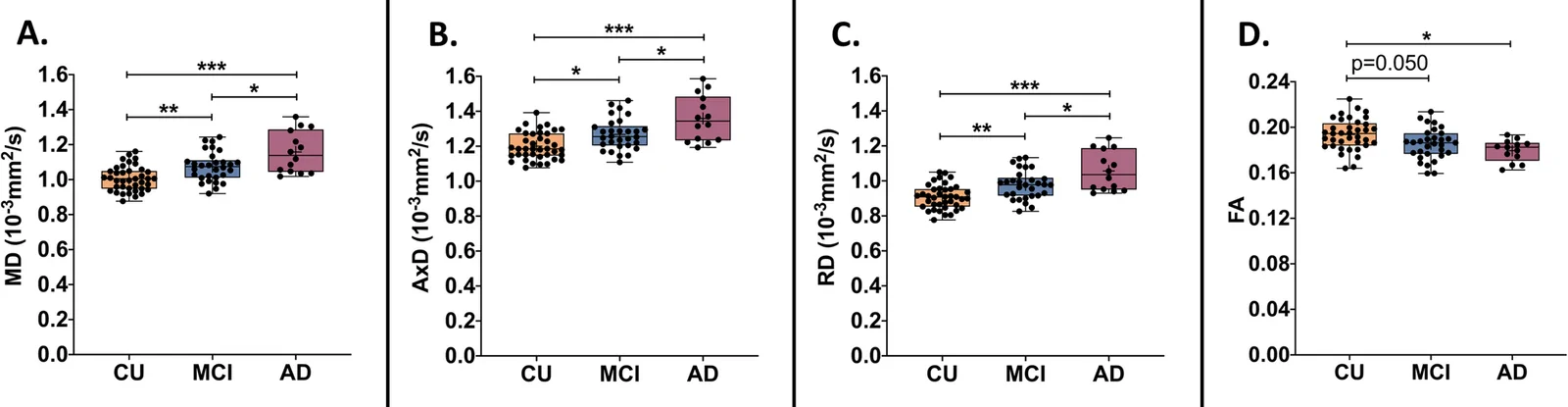
In vivo MRI diffusion differences in the left medial temporal lobe across diagnostic groups. **A** Mean diffusivity (MD). **B** Axial diffusivity (AxD). **C** Radial diffusivity (RD). **D** Fractional anisotropy (FA). *CU* Cognitively unimpaired individuals, *MCI* Mild cognitive impairment, *AD* Alzheimer’s Disease. Boxes represent the median and interquartile range, crosses indicate the mean, dots represent individual subjects. Statistical significance was assessed using ANCOVA, followed by Tukey post hoc comparisons. Statistical significance is indicated as *p < 0.05, **p < 0.01, ***p < 0.001

Regarding diffusion metrics, ANCOVA demonstrated a significant main effect of diagnostic group on MTL MD (F (2, 78) = 13.81, *p* < 0.001, partial η² = 0.26). Post-hoc comparisons of estimated marginal means with Tukey correction revealed progressively higher MD values from controls to MCI and AD. Specifically, AD patients showed a higher MD compared with controls (*p* < 0.001, Cohen’s *d* (*d*) = 1.628, CI [0.934, 2.322]) and MCI individuals (*p* = 0.031, *d* = 0.857, CI [0.183, 1.531]). MCI subjects also exhibited increased MD relative to controls (*p* = 0.006, *d* = 0.771, CI [0.268, 1.273]) (Fig. 7A).

A similar pattern was observed for AxD, with a significant effect of group (F (2, 78) = 12.36, *p* < 0.001, partial η² = 0.24). AxD values were higher in AD patients compared with both controls (*p* < 0.001, *d* = 1.557, CI [0.867, 2.247]) and MCI subjects (*p* = 0.029, *d* = 0.867, CI [0.192, 1.541]), and were also elevated in MCI individuals relative to controls (*p* = 0.017, *d* = 0.690, CI [0.190, 1.190]) (Fig. 7B). For radial diffusivity, there was also a significant group effect (F (2, 78) = 14.32, *p* < 0.001, partial η² = 0.27), with post-hoc comparisons showing increased RD in AD compared with both controls (*p* < 0.001, *d* = 1.648, CI [0.952, 2.343]) and MCI (*p* = 0.035, *d* = 0.842, CI [0.168, 1.516]), as well as higher values in MCI compared with controls (*p* = 0.004, *d* = 0.806, CI [0.302, 1.310]) (Fig. 7C).

In contrast, FA showed a significant main effect of group (F (2, 76) = 4.69, *p* = 0.012, partial η² = 0.11), but with a different pattern. FA was reduced in AD patients compared with controls (*p* = 0.028, *d* = -0.883, CI [-1.568,-0.199]), whereas no significant difference was observed between MCI and AD (*p* = 0.657, *d* = 0.298, CI [-0.381, 0.978]). A trend toward significance with lower FA in MCI relative to control was also found for this metric (*p* = 0.050, *d* = -0.585, CI [-1.083, -0.088]) (Fig. 7D).

To determine whether the observed diffusion abnormalities were independent of MTL atrophy, MTL volume was included as an additional covariate in the ANCOVA models. The overall pattern remained largely unchanged for MD (F(2,76) = 12.11, *p* < 0.001, partial η² = 0.24), AxD (F(2,76) = 10.39, *p* < 0.001, partial η² = 0.22), and RD (F(2,76) = 12.86, *p* < 0.001, partial η² = 0.25). For all three metrics, post hoc comparisons continued to show significantly higher diffusivity in both the MCI and AD groups compared with cognitively unimpaired controls (MD: MCI vs. CU, *p* = 0.004, *d* = 0.815, CI [0.304, 1.325]; AD vs. CU, *p* < 0.001, *d* = 1.522, CI [0.810, 2.234]; AxD: MCI vs. CU, *p* = 0.012, d = 0.730, CI [0.223, 1.237]; AD vs. CU, *p* < 0.001, *d* = 1.425, CI [0.719, 2.132]; RD: MCI vs. CU, *p* = 0.003, *d* = 0.852, CI [0.340, 1.364]; AD vs. CU, *p* < 0.001, *d* = 1.559, CI [0.845, 2.273]). However, the differences between the AD and MCI groups were no longer statistically significant for any of these metrics (MD: *p* = 0.109, *d* = 0.707, CI [0.009, 1.405]; AxD: *p* = 0.117, *d* = 0.695, CI [-0.003, 1.393]; RD: *p* = 0.109, *d* = 0.707, CI [0.009, 1.405]).

FA also remained significantly different among groups after volume adjustment (F(2,74) = 5.54, *p* = 0.006, partial η² = 0.13). FA was reduced in AD patients compared with controls (*p* = 0.019, *d* = -0.959, CI [-1.665, -0.252]); MCI participants also showed significantly lower values than controls (*p* = 0.024, *d* = -0.665, CI [-1.171, -0.160]), whereas the difference between MCI and AD remained non-significant (*p* = 0.679, *d* = 0.293, CI [-0.403,0.990]).

Overall, these findings indicate that the increased diffusivity observed in MCI and AD compared to controls is largely independent of MTL atrophy. In contrast, the disappearance of differences between MCI and AD after volume adjustment suggests that the additional diffusivity increase observed in AD (relative to MCI) is largely explained by volumetric loss. For FA, adjustment for MTL volume strengthened the difference between MCI and controls, indicating that volumetric changes did not account for the observed reduction in FA.

#### Comparison between in vivo and *postmortem* volumetric and diffusion-weighted MRI metrics

**Fig. 8:**
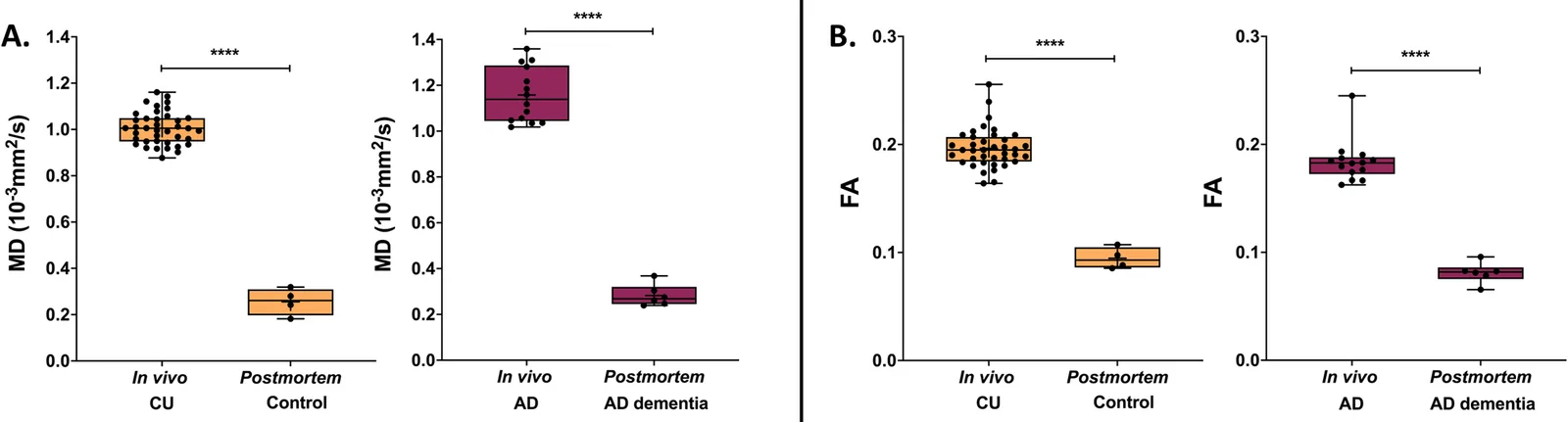
Comparison of in vivo and *postmortem* diffusion MRI parameters in the left medial temporal lobe between control and Alzheimer’s disease groups. **A** Mean diffusivity (MD). **B** Fractional anisotropy (FA). Boxes represent the median and interquartile range, crosses indicate the mean, dots represent individual subjects. Group comparisons were performed using independent-samples t-tests. Statistical significance is indicated as ****p < 0.0001

To determine whether diffusion MRI metrics exhibit systematic differences between in vivo and *postmortem* acquisitions, we compared MTL diffusion measures obtained from both datasets. This comparison aimed to provide context for interpreting *postmortem* MRI findings in relation to clinically acquired in vivo imaging. For MD, values were markedly lower in *postmortem* data compared with in vivo measurements; this applied to comparisons both within control groups (mean difference = − 0.750, CI [− 0.824, − 0.676], *t*(41) = 20.46, *p* < 0.0001, Hedges’ *g* = 10.54) and AD groups (mean difference = − 0.876, CI [− 0.982, − 0.769], *t*(18) = 17.25, *p* < 0.0001, Hedges’ *g* = 8.06), (Fig. 8A).

Similar to MD, FA values were reduced in *postmortem* measurements relative to in vivo imaging in both control groups (mean difference = − 0.102, CI [− 0.121, − 0.083], *t*(41) = 10.96, *p* < 0.0001, Hedges’ *g* = 5.65) and AD groups (mean difference = − 0.103, CI [− 0.121, − 0.085], *t*(18) = 12.01, *p* < 0.0001, Hedges’ *g* = 5.61), (Fig. 8B)

 These findings demonstrate that absolute diffusion values differ substantially between in vivo and *postmortem* imaging, consistent with known effects of tissue fixation and acquisition conditions. Therefore, *postmortem* diffusion metrics should be interpreted primarily in terms of their biological associations with tissue microstructure rather than by direct comparison of their absolute values with in vivo measurements

## Discussion

In this study, we associated *postmortem* MRI with cellular and neuropathological findings to investigate the microstructural substrates of medial temporal lobe alterations in dementia and cognitively unimpaired subjects. The *postmortem* strategy—applied in the same subjects—showed that most diffusion MRI metrics displayed negative correlations with quantitative cellular composition in the MTL and positive correlations with the neuropathological burden in subjects with AD. The in vivo approach—applied in a different cohort—showed that AD patients exhibited reduced MTL volume and Fractional Anisotropy, with an increase of other diffusion MRI metrics (Mean, Axial and Radial Diffusivity). Together, these findings provide converging evidence that diffusion MRI metrics are sensitive to cellularity alterations and vary across the clinical progression of AD.

### Diffusion MRI metrics are associated to cellular loss and neuropathological burden

Alterations in MRI parameters depend on the local biophysical environment of hydrogen protons in tissue, which are primarily associated with water molecules (Basser and Jones 2002; Soares et al. 2013). However, the specific cellular and tissue-level substrates underlying these signals remain incompletely understood. *Postmortem* MRI is a valuable tool to compare its metrics with brain histology under high resolution. We performed the *postmortem* acquisitions without time constraints – more than 3 h for each brain hemisphere – aiming to investigate the relationship between MRI volumetric and diffusion parameters, quantitative cellular measures (absolute cell counts), and neuropathological findings associated with dementia.

In the *postmortem* sample, group differences were observed for the MTL number of neurons and volume, with lower values in AD subjects related to controls. In contrast, no statistically significant group differences were observed for diffusion MRI metrics. This lack of group-level effects is likely attributable, at least in part, to the small sample size per group, resulting in limited statistical power to detect subtle differences. In addition, in the *postmortem* subsample imaged, the control group exhibited higher values of Braak (≥ IV) and CERAD scores. This could reduce between-group differences in *postmortem* MRI measures. The heterogeneity of NP findings and the variability in cellular measures enabled a broader dynamic range for subsequent correlation analyses with volumetric and diffusion MRI parameters.

We observed a trend toward a positive association between volume and absolute cell number in the MTL. Additionally, diffusion MRI metrics (MD, AxD, and RD) showed moderate but significant negative correlations with total cell counts, indicating that increased water diffusivity is associated with reduced tissue cellularity. Although significant associations were observed between cellularity and imaging parameters, a predictive model of cellularity from MRI measures was not robust, likely due to limited sample size and biological complexity. More detailed cellular features, such as cell size, synaptic density, and additional extracellular matrix components, were not assessed and may also contribute to diffusion MRI changes.

Diffusion metrics also correlated with established neuropathological staging systems in AD, including Braak and CERAD scores, suggesting that diffusion MRI alterations not only reflect cell loss, but are linked to the degree of AD pathology. These findings are consistent with recent clinicopathological studies showing that in vivo diffusion MRI metrics are associated with AD neuropathological burden. In a clinicopathological study of 43 autopsy cases, Torso et al. (2023) reported significant associations between *antemortem* cortical diffusivity and AD neuropathological markers, including Thal phase, Braak stage, and CERAD neuritic plaque scores, predominantly in temporal and limbic regions. More recently, Alasmar et al. (2026) extended these findings by demonstrating that in vivo MRI-derived mean diffusivity reflects the severity of *postmortem* neuropathology and clinical progression. Notably, both studies reported positive associations between higher cortical MD values and Braak stage, which is in line with our result. Importantly, our findings extend this literature by providing direct evidence that these diffusion abnormalities are associated with quantitative reductions in absolute tissue cellularity, supporting the biological interpretation that diffusion MRI reflects the underlying microstructural degeneration accompanying AD pathology.

Imaging tools may represent valuable biomarkers for early diagnosis and monitoring, as they reflect underlying microstructural processes related to cellular composition changes and neuropathological burden.

### Diffusion MRI metrics in the medial temporal lobe vary with the clinical progression of Alzheimer’s disease

Consistent with previous literature, we found reduced MTL, hippocampal, and other subregional volumes in AD patients, while MCI individuals did not differ from controls. These findings show that atrophy becomes more prominent at later stages of disease progression. Conventional MRI is widely used in patients with cognitive decline and/or suspected Alzheimer’s disease to detect macrostructural changes (Gu et al. 2010), such as hippocampal atrophy, cerebral cortex thinning, and lower brain volume (Kamatham et al. 2024). These results indicate that volumetric and thickness reduction become prominent at the dementia stage of Alzheimer’s disease.

In addition, diffusion metrics differed already at the MCI stage, showing progressively higher values (for MD, AxD and RD) across diagnostic groups. These findings reinforce the potential of diffusion metrics, particularly MD and its directional components, as early markers of AD-related microstructural disruption in gray matter. Studies in patients with autosomal dominant Alzheimer’s disease have demonstrated that DWI is useful for assessing changes in cortical and subcortical diffusivity that begin before the onset of symptoms (Weston et al. 2015). Our results suggest that diffusion MRI is sensitive to microstructural alterations that precede atrophy, with a gradual increase in diffusivity across the AD spectrum. In the sporadic form of the disease, previous work has similarly shown that grey matter MD changes precede macroscopic alterations such as volume loss (Weston et al. 2015), and increased MD is associated with Aβ deposition (Debatisse et al. 2025).

The diagnostic accuracy of MD has been used to differentiate patients with dementia from those with MCI and cognitively unimpaired controls. Brueggen and colleagues (2018) found that right and left hippocampal MD changes were sensitive to predicting dementia. In fact, MD sensitivity was greater than volume to differentiate these diagnostic groups. Diffusion alterations have also been reported in other neurodegenerative conditions, such as Parkinson’s disease, with observed MD differences compared to CUs in caudate, putamen, globus pallidus, and thalamus, as well as in various white matter tracts such as the corpus callosum, uncinate, cingulate, superior longitudinal fasciculi, and thalamic radiation (Kim et al. 2013).

Future investigations integrating additional histological techniques with higher-resolution diffusion models and including individuals at earlier stages of the Alzheimer’s disease continuum may further refine the biological interpretation of these metrics in this context. Diffusion abnormalities may reflect additional microstructural changes beyond those evaluated in the present study. These may include glial alterations, dendritic degeneration, synaptic loss, and changes in the extracellular space, all of which could contribute to the diffusion MRI signal.

### Methodological considerations for the comparison of In Vivo and Postmortem MRI

In addition to the significant correlations discussed above, although *postmortem* imaging lacks direct clinical diagnostic utility, its primary value lies in the ability to validate the biological substrates of in vivo signals. It opens the way, however, to complement the correlation analyses by including imaging metrics obtained in vivo. We observed systematic differences between in vivo and *postmortem* MRI measures, including reduced MD, FA, AxD, and RD in *postmortem* tissue. These findings are consistent with prior studies (Schilling et al. 2025; Neuhaus et al. 2025) and likely reflect physicochemical changes associated with the *postmortem* state.

The results support previous recommendations to consider chemical and physical variables when performing *postmortem* MRI (Schilling et al. 2025). After death, brain tissue undergoes autolysis followed by bacterial degradation, resulting in a gradual tissue decomposition (D’Arceuil and de Crespigny 2007). Over time, protein denaturation and increased enzymatic activity promote tissue and cellular liquefaction, along with other chemical changes, including pH alterations that may compromise the integrity of proteins and nucleic acids (Blair et al. 2016). These alterations are usually arrested with the use of chemical fixation, which preserves cells and other constituents of the tissue, stopping autolysis and bacterial decomposition, and thus providing a closer picture of the in vivo state, guaranteeing the preservation of the tissue architecture and the possibility of performing histological and pathological studies (Dawe et al. 2009). *Postmortem* interval (PMI, time between death and fixation) should be considered when performing imaging and must be minimized (Schilling et al. 2025). MRI is also susceptible to noise due to MRI delay (time between death and MRI); thus, this variable must be also rigorously controlled.

After immersion, fixative molecules progressively diffuse from the outer surface to the deepest parts of the tissue, causing protein cross-linking and therefore reducing diffusion speed along time. The changes in MRI parameters can be secondary to the speed of replacement rate between free and restricted water, tissue dehydration, and the substitution of physiological fluids by the fixative (Shatil et al. 2018). Additionally, temperature is also a significant physical factor that influences MRI (Berger et al. 2022). T2 and ADC can vary due to changes between 10 and 37 °C (Thelwall et al. 2006). More detailed information on the factors influencing *postmortem* MRI is provided in Supplementary Material S3.

The variations in acquisition protocols, including MRI scanner type and sequence parameters, likely contributed to the observed differences in our results. The diffusion voxel size differed between the *postmortem* (1.5 mm isotropic) and in vivo (2.5 mm isotropic) imaging protocols. This ∼4.6-fold difference in voxel volume represents an inherent trade-off in cross-modality validations; the absence of motion artifacts and the extended scanning windows available *postmortem* allow high-resolution imaging with robust SNR. Conversely, in vivo scans are strictly limited by scan time constraints to minimize patient fatigue and motion, requiring a larger voxel to maintain an acceptable baseline SNR. While the higher spatial resolution of the *postmortem* data minimizes partial volume effects, particularly at tissue interfaces (e.g., grey/white matter junctions or near the CSF), the in vivo data inherently experience greater spatial smoothing.

Furthermore, the adjusted b-values across both cohorts were intentionally selected to account for the biophysical alterations induced by fixation. Because chemical fixation significantly restricts the mobility of water molecules, reducing the baseline diffusion coefficient by 50–70%, higher b-values are methodologically required in fixed specimens to achieve signal attenuation and angular contrast comparable to living tissue (Schilling et al. 2025).

Another methodological remark should be considered concerning our study. Global neuropathological diagnosis (including Braak staging and CERAD scoring) was performed in the right hemisphere, whereas MRI acquisition and cellular quantification were conducted in the left hemisphere. This design is standard in brain banking protocols (Shepherd et al. 2019), where neuropathological staging is routinely established from a single fixed hemisphere. It was also operationally necessary because the fixation and processing requirements for optimal histological staining differ from those best suited to isotropic fractionator-based cell counting, which requires complete tissue homogenization.

This protocol assumes a degree of interhemispheric symmetry in AD-related pathology. AD is generally considered a bilateral neurodegenerative process with symmetrically distributed pathology (Giannakopoulos et al. 2009), supporting the use of contralateral hemispheres as a reasonable approximation of an individual’s overall pathological stage. This does not exclude the possibility of regional hemispheric asymmetries; indeed, subtle differences in neuropathological burden and microstructural degeneration have been reported, particularly in early disease stages and in atypical AD variants (e.g., posterior cortical atrophy, logopenic variant primary progressive aphasia) (Tremblay et al. 2022; Lu et al. 2023; Anijärv et al. 2025).

Our design of the *postmortem* study ensured that imaging metrics and absolute cell counts were directly correlated within the same hemisphere. Consequently, assigning global pathological scores derived from the contralateral hemisphere may represent a potential limitation. Possible hemisphere-specific differences may have contributed to variability in the observed associations. However, any residual interhemispheric variability would be expected to weaken rather than strengthen the correlations reported here. Therefore, our estimates of the relationship between diffusivity, cellular loss, and neuropathological burden are likely conservative.

## Conclusions

In this study, we combined in vivo MRI, *postmortem* MRI, and quantitative histology to investigate the microstructural alterations of the human medial temporal lobe in dementia, with particular interest in Alzheimer’s disease. *Postmortem* analyses provided mechanistic grounding for imaging metrics through direct validation at the cellular level, and in vivo MRI demonstrated how these imaging findings relate to clinically meaningful disease progression. We found that increased diffusivity metrics (MD, AxD and RD) are associated with reduced cellularity and higher tau-related neuropathological burden, and FA with the neuritic plaques score. These results provide direct evidence that diffusion MRI alterations in the MTL reflect underlying tissue degeneration. The in vivo results demonstrate that volumetric reductions and increased diffusivity metrics within the MTL are prominent at the dementia stage of AD, with mean, axial, and radial diffusivity showing progressive changes across the cognitive continuum from controls to MCI and AD dementia. The observed differences between in vivo and *postmortem* diffusion values are consistent with known effects of death and tissue fixation, underscoring the need for cautious interpretation when translating *postmortem* MRI findings to living tissue.

Together, our findings strengthen the biological interpretability of diffusion MRI metrics in Alzheimer’s disease and highlight their potential as non-invasive markers of microstructural degeneration. By bridging in vivo imaging with *postmortem* cellular and pathological data, this work contributes to a more mechanistic understanding of MRI biomarkers, providing a biological basis for their potential utility in the study of disease progression in clinical AD.

## Supplementary Information

Below is the link to the electronic supplementary material.

Supplementary Material 1

## Data Availability

The data are available from the corresponding author upon reasonable request.

## Abbreviations

3T: 3 Tesla
AD: Alzheimer’s disease
ADC: apparent diffusion coefficient
AGD: Argyrophilic Grain Disease
AMY: Amygdala
ANCOVA: Analysis of covariance
AxD: Axial diffusivity
B0: Non-diffusion-weighted (b = 0) image/volume
Braak: Braak neurofibrillary tangle staging system
CDR: Clinical Dementia Rating
CERAD: Consortium to Establish a Registry for Alzheimer’s Disease
CONEP: Brazilian National Committee of Ethics in Research
CU: Cognitively unimpaired controls
DAPI: 4’,6-diamidino-2-phenylindole
DSM-5: Diagnostic and Statistical Manual of Mental Disorders, 5th edition
DTI: Diffusion tensor imaging
DTIFIT: FSL tool for diffusion tensor fitting
DWI: Diffusion-weighted imaging
EC: Entorhinal cortex
FA: Fractional anisotropy
FLIRT: FMRIB’s Linear Image Registration Tool (FSL)
FOV: Field of view
FTLD: Frontotemporal lobar degeneration
FSL: FMRIB Software Library
GM: Gray matter
HIV: Human immunodeficiency virus
HPC: Hippocampus
IDOR: D’Or Institute for Research and Education
IQR: Interquartile range
JHU: Johns Hopkins University
LATE: Limbic-predominant age-related TDP-43 encephalopathy
LBD: Lewy body disease
MCI: Mild cognitive impairment
MD: Mean diffusivity
MNI: Montreal Neurological Institute
MRI: Magnetic resonance imaging
MPRAGE: Magnetization-prepared rapid gradient-echo
MTL: Medial temporal lobe
NeuN: Neuronal nuclei marker
NGS: Normal goat serum
NP: Neuropathological/neuropathology
OOB: Out-of-bag (bootstrap evaluation set)
PBS: Phosphate-buffered saline
PFA: Paraformaldehyde
PHC: Parahippocampal cortex
PMI: Postmortem interval
PSP: Progressive supranuclear palsy
PTE: Pulmonary thromboembolism
R²: Coefficient of determination
RD: Radial diffusivity
RMSE: Root mean squared error
ROI: Region of interest
RS: Rhinal sulcus
SD: Standard deviation
SSres: Residual sum of squares
SStot: Total sum of squares
TDP-43: TAR DNA-binding protein 43
TE: Echo time
TR: Repetition time
VaD: Vascular dementia
WM: White matter
χ²: Chi-square test statistic
