# Supplementary material for: Microstructural alterations of human medial temporal lobe in dementia: volumetric and DTI findings correlate with cell number and neuropathology
Source: Brain Struct Funct. 2026 Nov 13;231(9):167. doi: 10.1007/s00429-026-03223-4 (PMC13768387; doi:10.1007/s00429-026-03223-4)
Supplement: Supplementary file 1 [file 429_2026_3223_MOESM1_ESM.docx]

**Supplementary Material**

**Supplementary Material S1. Clinical and demographic variables of brain donors**

**Table S1**. Clinical and demographic variables of brain donors

| Nº | Sex | Age (y) | CDR | Brain mass  (g) | PMI  (h) | Cause  of  death | Braak  AD  stage | CERAD  AD  score | AD+ | Other NP  findings | Fixation  Time  (h) | MRI  Delay (days) |
| --- | --- | --- | --- | --- | --- | --- | --- | --- | --- | --- | --- | --- |
| 1 | F | 71 | 0 | 1094 | 15 | PTE | IV | None | No | Normal,  AGD | 39 | 34 |
| 2 | F | 79 | 0 | 1057 | 10 | Acute  pancreatitis | V | Moderate | AD | VaD | 37 | 28 |
| 3 | F | 84 | 0 | 1084 | 14 | Hemopericardium | IV | None | No | Normal,  AGD | 39 | 29 |
| 4 | F | 87 | 0 | 976 | 15 | Pulmonary  edema | IV | None | No | Normal,  AGD | 39 | 29 |
| 5 | F | 87 | 0 | 1145 | 15 | PTE | V | Moderate | AD |  | 39 | 40 |
| 6 | M | 65 | 0 | 1413 | 25 | Acute MI | IV | None | No | VaD,  AGD | 38 | 35 |
| 7 | M | 76 | 0 | 1275 | 17 | Bronchopneumonia | IV | None | No | Normal,  AGD | 36 | 33 |
| 8 | M | 85 | 0 | 1054 | 11 | Gallbladder  empyema | VI | Moderate | AD | VaD | 39 | 35 |
| 9 | M | 96 | 0 | 1117 | 10 | Pulmonary  edema | V | Mild | No | AGD,  PSP, VaD | 39 | 28 |
| 10 | F | 67 | 3 | 1194 | 18 | Pulmonary  edema | III | Moderate | AD |  | 36 | 40 |
| 11 | F | 77 | 3 | 1222 | 17 | Acute MI | III | Mild | No | VaD,  AGD | 36 | 27 |
| 12 | F | 82 | 3 | 970 | 7 | Sepsis | VI | Moderate | AD | AGD | 39 | 33 |
| 13 | F | 88 | 3 | 925 | 12 | Pulmonary  edema | V | Moderate | AD |  | 38 | 36 |
| 14 | F | 88 | 3 | 985 | 14 | Pulmonary  edema | VI | Moderate | AD | AGD,  VaD | 39 | 14 |
| 15 | F | 88 | 3 | 962 | 12 | Pulmonary  edema | VI | Moderate | AD | VaD,  PSP | 39 | 27 |
| 16 | F | 92 | 3 | 952 | 18 | Bronchopneumonia | IV | Moderate | AD | AGD,  VaD | 39 | 28 |
| 17 | M | 78 | 3 | 989 | 20 | Bronchopneumonia | VI | Severe | AD | AGD,  VaD | 37 | 21 |
| 18 | M | 82 | 3 | 1341 | 15 | Pulmonary  edema | V | None | No | AGD, LBD,  VaD | 40 | 20 |

*Abbreviations:* AD: Alzheimer’s Disease; AGD: Argyrophilic Grain Disease; h: hours, LBD: Lewy Body Disease; MI: Myocardial Infarction; NP: Neuropathological; PMI: postmortem interval; PSP: Progressive Supranuclear Palsy; PTE: Pulmonary thromboembolism; VaD: Vascular disease.

**Supplementary Material S2. Neuropathological assessment**

Exclusion criteria included individuals with a clinical history of neurological or psychiatric diseases, HIV infection diagnosis, alcohol or drug abuse, absence of a family member or caregiver who could provide adequate and reliable information, and acidosis (cerebrospinal fluid pH<6.5) that makes tissue incompatible for biochemical and molecular studies of the brain.

The list of sampled brain regions used for neuropathological assessment included the middle frontal gyrus, middle and superior temporal gyri, angular gyrus, superior frontal and anterior cingulate gyrus, visual cortex, hippocampal formation, amygdala, basal ganglia, thalamus, midbrain, pons, medulla oblongata, and cerebellum.

**Supplementary Material S3. *Postmortem* MRI acquisition**

Brain images were acquired at the D’Or Institute for Research and Education (IDOR) using a 3T Siemens magnet (Siemens Healthcare, Erlangen, Germany). Previous pilot tests to control for confounding variables and to improve signal-to-noise ratio were made in a 3T Philips Achieva MRI scanner (Philips Healthcare, Best, the Netherlands) in 10 cases, not included in the results but important for protocol optimization and artifact control.

Postmortem MRI is prone to artifacts, which were carefully controlled to ensure optimal image quality. The left hemisphere was immersed in PBS in a customized sample holder. The acquisition was performed in a temperature-controlled environment (21°C). Foam cushions were arranged underneath and around the sample holder to avoid vibration artifacts. Other factors were controlled, of which a detailed description can be found below.

**Fixation:** Following immersion, fixative molecules gradually diffuse from the outer toward the inside surface of the tissue, leading to gradual protein cross-linking. As the fixative penetrates deeper, diffusion slows, resulting in more effective fixation at the surface than in deeper regions. For this reason, intravascular perfusion is preferred in animal models. Both, the type of fixative and the duration of fixation can impact MRI images; the alterations in MRI parameters may be attributed to changes in water exchange rates between free and restricted compartments, tissue dehydration, and the replacement of physiological fluids by the fixative (Shatil et al. 2018). In this study, 2% PFA in PBS was used for all samples, with a controlled fixation time of 36 to 40 hours.

**Bubbles:** During pilot acquisitions, air bubbles were detected between cerebral sulci and within the lateral ventricles, impairing accurate delineation of gray matter boundaries. To mitigate this problem, the left hemisphere immersed in PBS was first let to reach room temperature prior to imaging. The container holding the hemisphere was then placed inside a larger (15L) PBS-filled container at room temperature, ensuring continuous contact between the tissue and the solution and minimizing air exposure during handling. The hemisphere was gently rotated within the liquid to facilitate the release of trapped air from sulcal and ventricular spaces, effectively preventing bubble formation during MRI acquisition.

**Immersion liquid:** All acquisitions were acquired in PBS as immersion fluid. No proton-free liquid was employed for this project.

**Recipient holder:** During pilot image acquisitions, a hypersignal near the base and GM-WM signal inversion were observed due to the plastic container. To address this, imaging was standardized using an airtight plastic bag filled with PBS. This setup ensured that the tissue remained floating, avoiding contact with any surface while staying fully immersed in PBS, with minimal space to limit movement during acquisition. The left hemisphere was positioned with its medial face at the bottom of the bag and the lateral surface oriented upwards.

**Temperature:** T2 and ADC values can vary due to temperature fluctuations between 10°C and 37°C (Thelwall et al. 2006). Therefore, maintaining a standardized temperature is essential to minimize variability in relaxation times and diffusion measurements. Therefore, all acquisitions were conducted in the MRI room, maintained at a constant temperature of 21°C.

***Postmortem* MRI preprocessing and ROI registration**

MTL Region of Interest (ROI) included nine subregions identified in the atlas available in the FSL: Hippocampus and amygdala [Harvard-Oxford structural subcortical atlas], anterior and posterior parahippocampal gyrus [Cortical atlas Harvard-Oxford cortical], cornu ammonis, subiculum, entorhinal, cingulate-hippocampal, lateral and superficial amygdala [Jülich Histological Atlas (cyto and myelo-architecture)], cingulate-hippocampus [Atlas of white matter based on DTI JHU].

**Supplementary Material S4. MTL dissection and cell quantification by isotropic fractionation**

The medial temporal lobe was delimited by an incision following the border of the uncus, the rhinal and collateral sulci. The posterior border was set by a coronal section passing through the most caudal level of the splenium of the corpus callosum (Figs. 1F, G, H). This level usually coincides with the calcarine sulcus rostral pole, which defines the parahippocampal gyrus caudal border (Van Hoesen 1995). The lateral border was the wall of the lateral ventricle. The medial temporal lobe includes sub-regions such as the uncus, amygdala, hippocampus, subiculum, pre-subiculum, and parahippocampal gyrus. Our group has previously standardized and published this dissection scheme (Andrade-Moraes et al. 2013; Oliveira-Pinto et al. 2016; de Morais et al. 2023).

For the isotropic fractionation, a chemomechanical dissociation was performed by placing the samples in a solution containing 40 mM Sodium Citrate plus 1% Triton X-100 and homogenized in a semi-automated machine (Fig. 2I) (Azevedo et al. 2013). After the dissociation process, the anatomical structures of complex cytoarchitecture were converted into a homogeneous (isotropic) suspension of nuclei (Figs 1G, 2J), which was maintained homogeneous by continuous agitation. The final volume of the suspension was calculated, and four aliquots of 10 μl were taken for counting.

The absolute number of cells was obtained by counting the density of nuclei identified with a 2% DNA intercalant (4’,6-diamidino-2-phenylindole dihydrochloride, DAPI, Sigma-D9542, 20mg/L) solution, disposed on a hemocytometer (Fig. 1H) and visualized at the fluorescence microscope (Zeiss AxioImager, 20X) (Herculano-Houzel and Lent 2005). The coefficients of variation among samples were less than 15%. The average density was calculated to obtain the total number of cells, assuming that all neural cells have one and only one nucleus.

Neurons were identified by performing immunocytochemical labeling of NeuN (a splicing factor, Fox3) (Mullen et al. 1992; Wolf et al. 1996; Kim et al. 2009). Two samples of 1000µL from the nuclei suspension were collected, centrifuged, and washed with PBS. One sample was incubated with the primary antibody anti-mouse NeuN (MAB377, Millipore) 1:200 in PBS at 4°C overnight, and the other sample was used as a negative control. A secondary reaction was performed on the next day with the secondary antibody (Alexa-Fluor 546, Thermo-Fisher, A11003, at 1:300 PBS in a solution containing 10% NGS and 3% DAPI) for two hours at 4°C. The proportion of neurons was determined from at least 500 DAPI-positive nuclei. Absolute numbers of neurons and non-neuronal cells were extrapolated to the whole suspension volume for each tissue sample.

**Supplementary Material S5. *In vivo* outliers.**

***Table S2*.** Outliers identified and excluded from the corresponding analyses in the *in vivo* cohort.

| MRI measure | CU  group | MCI  group | AD  group | Total |
| --- | --- | --- | --- | --- |
| Left entorhinal cortex thickness | 3 | 1 | 2 | 6 |
| Right entorhinal cortex volume | 2 | 0 | 0 | 2 |
| Left entorhinal cortex volume | 1 | 1 | 0 | 2 |
| Right amygdala volume | 1 | 2 | 0 | 3 |
| Left medial temporal lobe volume | 1 | 3 | 0 | 4 |
| FA | 2 | 0 | 1 | 3 |
| MD | 0 | 1 | 0 | 1 |
| AxD | 0 | 1 | 0 | 1 |
| RD | 0 | 1 | 0 | 1 |

*Abbreviations:* AD: Alzheimer's disease; AxD: axial diffusivity; CU: cognitively unimpaired; FA: fractional anisotropy; MCI: mild cognitive impairment; MD: mean diffusivity; RD: radial diffusivity.

**Supplementary Material S6. *Cellularity prediction model***

***Cellularity prediction model methods.*** To investigate the association between *postmortem* MRI-derived metrics and total cellular count, we fitted two regression-based predictive models: a standard multiple linear regression model and a ridge regression model with L2 regularization. The outcome variable was total cell count, scaled to absolute units (x 10⁶), and predictors included left medial temporal lobe volume and mean diffusivity, with diffusivity values rescaled (x 10⁻³) for numerical stability.

Model performance and parameter uncertainty were assessed using a nonparametric bootstrap framework with 10,000 resamples. For each bootstrap iteration, models were trained on a resampled dataset and evaluated on the corresponding out-of-bag (OOB) observations (i.e., subjects not included in the resampled set). Predictive accuracy was quantified using the root mean squared error (RMSE) and the out-of-bag coefficient of determination (R²), defined as:

$$R^{2}=1-\frac{{SS}_{res}}{{SS}_{tot}}$$

$SS_{res}$ denotes residual sum of squares and $SS_{tot}$the total sum of squares of the OOB sample. Bootstrap distributions were used to estimate mean performance, standard deviations, and 95% percentile confidence intervals.

Ridge regression models were implemented using the *glmnet* framework with α = 0 (pure L2 penalty). The regularization parameter (λ) was selected via five-fold cross-validation on the full dataset, minimizing mean squared error. The optimal λ was then held constant across bootstrap iterations. Differences in predictive performance between linear and ridge regression were quantified by computing bootstrap distributions of the RMSE and R² differences across paired resamples. All analyses were implemented in R (v. 4.4.2).

***Cellularity prediction model results.*** The bootstrap evaluation (10,000 resamples) of the multiple linear regression model revealed limited predictive performance. The mean out-of-bag RMSE was 181 x 10^6^ cells (SD = 43 x 10^6^), with a 95% percentile interval ranging from 102 x 10^6^ to 268 x 10^6^. The mean out-of-bag R² was negative (mean = −0.83, SD = 17.52), with a wide 95% interval spanning from −4.53 to 0.52. The predominance of negative R² values indicates that, in most bootstrap iterations, the model performed worse than a null model predicting the mean cell count of the out-of-bag sample. Bootstrap confidence intervals for regression coefficients also demonstrated substantial instability. The 95% percentile intervals were: -8.13 x 10^6^ to 5.30 x10^7^ for the volume, and -2.33 x 10^12^ to 1.85 x 10^11^ for the mean diffusivity.

The ridge regression model demonstrated modestly improved predictive stability compared to the ordinary linear regression. The mean out-of-bag RMSE was 171 x 10^6^ cells (SD = 29.6 x 10^6^), with a 95% percentile interval between 115 x 10^6^ and 233 x 10^6^. Notably, the variability of RMSE across bootstrap iterations was lower than that observed for the linear model. The mean out-of-bag R² remained negative (mean = −0.40, SD = 2.04), with a 95% interval ranging from −3.15 to 0.34. Although still predominantly below zero, the ridge model exhibited reduced dispersion in R² estimates compared to the linear regression, indicating greater numerical stability across resamples. Similar to what was observed in the linear regression model, confidence intervals for the ridge coefficients showed instability. The 95% percentile intervals were: -7.72 x 10^6^ to 5.23 x10^7^ for the volume, and -2.33 x 10^12^ to 1.71 x 10^11^ for the mean diffusivity. Direct bootstrap comparison of performance metrics indicated a small average reduction in RMSE for ridge regression relative to linear regression (~ 10 x 10^6^ cells).

Our findings indicate that *postmortem* MRI-derived measures of left medial temporal lobe volume and mean diffusivity were not robust predictors of total cellular count under a bootstrap out-of-bag validation framework. Across 10,000 resamples, both linear and ridge regression models exhibited predominantly negative R² values and high RMSE, indicating that predictions frequently performed worse than a null model based solely on the sample mean. Although ridge regression reduced variance and slightly improved average RMSE, this gain reflected increased numerical stability rather than meaningful predictive improvement. Thus, while regularization reduced variance, it did not substantially improve overall predictive accuracy. Future studies with larger samples, multimodal predictors, nonlinear modeling approaches, or region-specific cellular subtyping may be necessary to devise a robust model to predict the number of cells using MRI biomarkers.

**Supplementary References**

Andrade-Moraes CH, Oliveira-Pinto AV, Castro-Fonseca E, et al (2013) Cell number changes in Alzheimer’s disease relate to dementia, not to plaques and tangles. Brain 136:3738–3752. https://doi.org/10.1093/brain/awt273

Azevedo FAC, Andrade-Moraes CH, Curado MR, et al (2013) Automatic isotropic fractionation for large-scale quantitative cell analysis of nervous tissue. J Neurosci Methods 212:72–78. https://doi.org/10.1016/j.jneumeth.2012.09.015

de Morais VAC, de Oliveira-Pinto AV, Mello Neto AF, et al (2023) Resilience of Neural Cellularity to the Influence of Low Educational Level. Brain Sci 13(1):104. https://doi.org/10.3390/brainsci13010104

Herculano-Houzel S, Lent R (2005) Isotropic Fractionator: A Simple, Rapid Method for the Quantification of Total Cell and Neuron Numbers in the Brain. J Neurosci 25:2518–2521. https://doi.org/10.1523/JNEUROSCI.4526-04.2005

Kim KK, Adelstein RS, Kawamoto S (2009) Identification of Neuronal Nuclei (NeuN) as Fox-3, a New Member of the Fox-1 Gene Family of Splicing Factors. J Biol Chem 284:31052–31061. https://doi.org/10.1074/jbc.M109.052969

Mullen RJ, Buck CR, Smith AM (1992) NeuN, a neuronal specific nuclear protein in vertebrates. Development 116:201–211. https://doi.org/10.1242/dev.116.1.201

Oliveira-Pinto AV., Andrade-Moraes CH, Oliveira LM, et al (2016) Do age and sex impact on the absolute cell numbers of human brain regions? Brain Struct Funct 221:3547–3559. https://doi.org/10.1007/s00429-015-1118-4

Shatil AS, Uddin MN, Matsuda KM, Figley CR (2018) Quantitative Ex Vivo MRI Changes due to Progressive Formalin Fixation in Whole Human Brain Specimens: Longitudinal Characterization of Diffusion, Relaxometry, and Myelin Water Fraction Measurements at 3T. Front Med 5:1–15. https://doi.org/10.3389/fmed.2018.00031

Thelwall PE, Shepherd TM, Stanisz GJ, Blackband SJ (2006) Effects of temperature and aldehyde fixation on tissue water diffusion properties, studied in an erythrocyte ghost tissue model. Magn Reson Med 56:282–289. https://doi.org/10.1002/mrm.20962

Van Hoesen GW (1995) Anatomy of the medial temporal lobe. Magn Reson Imaging 13:1047–1055. https://doi.org/10.1016/0730-725X(95)02012-I

Wolf HK, Buslei R, Schmidt-Kastner R, et al (1996) NeuN: A useful neuronal marker for diagnostic histopathology. J Histochem Cytochem 44:1167–1171. https://doi.org/10.1177/44.10.8813082
